# Temporal binding of interval markers

**DOI:** 10.1038/srep38806

**Published:** 2016-12-13

**Authors:** Christina Derichs, Eckart Zimmermann

**Affiliations:** 1 Institute for Experimental Psychology, Heinrich Heine University Düsseldorf, Universitätsstraße 1, 40225 Düsseldorf, Germany

## Abstract

How we estimate the passage of time is an unsolved mystery in neuroscience. Illusions of subjective time provide an experimental access to this question. Here we show that time compression and expansion of visually marked intervals result from a binding of temporal interval markers. Interval markers whose onset signals were artificially weakened by briefly flashing a whole-field mask were bound in time towards markers with a strong onset signal. We explain temporal compression as the consequence of summing response distributions of weak and strong onset signals. Crucially, temporal binding occurred irrespective of the temporal order of weak and strong onset markers, thus ruling out processing latencies as an explanation for changes in interval duration judgments. If both interval markers were presented together with a mask or the mask was shown in the temporal interval center, no compression occurred. In a sequence of two intervals, masking the middle marker led to time compression for the first and time expansion for the second interval. All these results are consistent with a model view of temporal binding that serves a functional role by reducing uncertainty in the final estimate of interval duration.

How do we determine the duration of temporal intervals? Strong illusions in time perception demonstrate a surprising plasticity of temporal interval estimations. For instance, the rapid movements of our eye, i.e. saccades, which compress apparent time^1^. The duration of brief intervals presented around onset of a saccade is drastically underestimated when compared with an interval that is observed without intervening saccade. Similar effects have been observed with covert attention shifts^2^. Saccades can also produce time expansion, a phenomenon known as “the stopped clock illusion”^3^. If a saccade is executed to the hand of a clock, the first second that follows the eye movement appears to last longer than the next. We have recently demonstrated that masking produces compression of temporal interval perception^4^. A whole-field texture mask presented in temporal vicinity of the interval marker led to an underestimation of the interval duration. For saccadic as well as for masking-induced time compression, we found that visual feature correspondence of the interval markers determined compression magnitude. When the interval markers differed in orientation, no compression occurred. The same dependency of compression on feature correspondence was also found in a multisensory setup^5^. What these illusions have in common is that two identical stimuli, or the on- and offset of a single stimulus, define a temporal interval. One of the interval markers falls into either the period of an action or an attention shift or it is masked, thus having a weak onset signal. We argued that temporal compression the outcome of a mechanism which acts against the variability of the weak onset signal of one of the interval markers that is produced by masking or the absence of attention^5^. A mask necessarily reduces the contrast of the interval marker. However the marker is still highly visible and therefore the reduction in contrast is an unlikely explanation for time compression. Instead, we have shown that distraction of spatial attention produces similar effects as the mask. Consistent with the literature^1^,^2^, we claimed that spatial attention drawn away from the interval marker is leading to the temporal uncertainty of that stimulus. We assumed that if one stimulus is temporally uncertain, the sensory system groups together those objects that correspond to each other. An analogy in the spatial dimension is the phenomenon of apparent motion^6^ where a postdictive analysis interprets smooth motion.

Why would grouping explain that intervals compress? Once the decision which markers correspond is made, the system estimates the time elapsed between those markers by summing their response distributions. Theories about neural time estimations assume that on- or offsets of stimuli are compared to a reference activity which either is explicitly given as a neural clock^7^ or implicitly embedded in neural activation^8^. Stimuli with weak onset signals will have broad, i.e. variable, response distributions. Thus, relating their temporal occurrence to the reference activity becomes more variable. By summing the neural distributions responding to the weak onset signal with that of the strong onset signal the variance in the final estimate will be minimized. The peak corresponding to the summed distribution will be shifted in the direction of the strong onset signal, resulting in duration compression for the interval between the two markers. Temporal compression therefore is the consequence of the systems’ attempt to provide meaning in dynamic visual scenes by matching corresponding objects. Temporal binding serves the functional role of a reduction in the variance of subjective interval durations. Similar arguments have been raised in the context of simultaneity illusions, where it has been claimed that marker correspondence and not neural processing latencies determines temporal binding of visual attributes^9^. This view rejects the idea that event time is inferred from brain time and instead suggests a postdictive estimation of time^10^,^11^.

In the present study we aimed to find evidence for temporal binding in interval estimations. To this end, we used a masking procedure that yields temporal interval compression^4^. We first asked whether masking affects the temporal registration of one of the interval markers. Interval compression might result from a change in the neural processing duration for an interval marker. In a temporal masking paradigm, we found that masking did not affect the temporal processing of the single stimuli. Changes in the processing speed of the masked stimulus would predict that interval estimations should differ when either the first or the second marker is masked. However, we found interval compression in both conditions. We next asked about the functional benefit of the putative temporal binding mechanism, i.e. a reduction in the thresholds for intervals in which one of the markers was presented in close temporal vicinity to a mask. Thresholds were lower when interval estimations were compressed, i.e. when one of the marker was masked. We also tested a sequence of two intervals. We presented three interval markers which defined two intervals. Masking the second marker led to a compression of the first and an expansion of the second interval. Masking the first and the second interval marker however, left estimations for both intervals unaffected. All these results are consistent with the idea that time compression results from temporal binding of interval markers.

## Results

**Figure 1:**
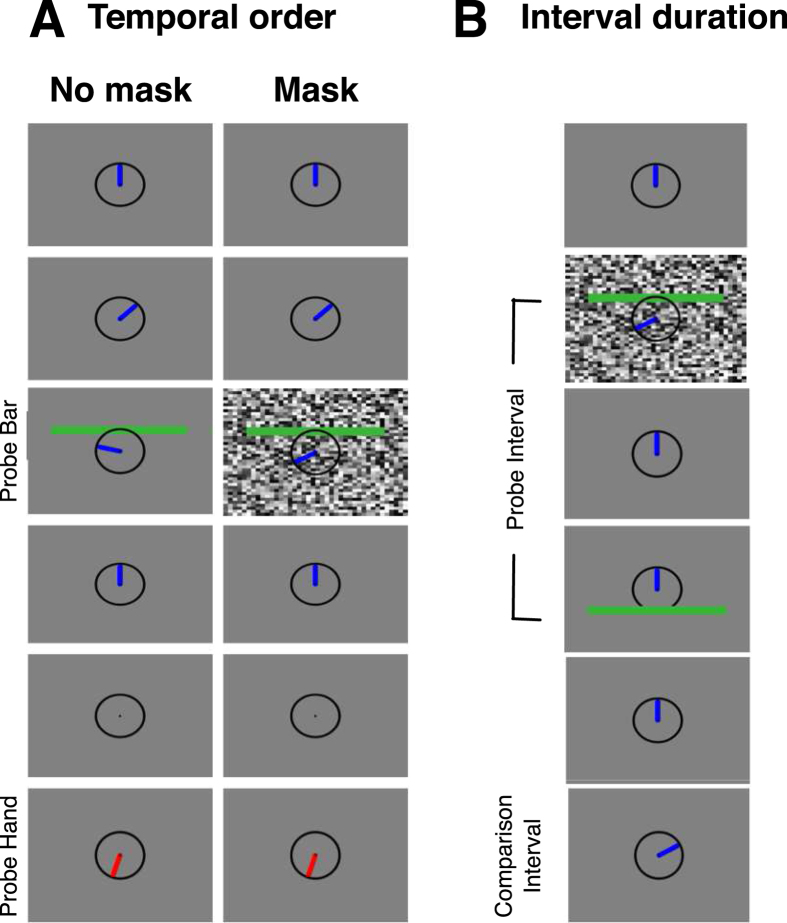
(**A**) Arrangement of stimuli in the temporal order judgment experiment (Experiment 1). A clock without ticks and a fixation point at its center were visible throughout the trial. While a blue hand made a full rotation a green bar (probe stimulus) was flashed. In the mask condition the probe was presented on top of a mask. After the clock hand finished turning a red hand (comparison stimulus) was presented 1500 ms later. Subjects had to judge whether the flashed probe stimulus occurred before or after the clock hand was in the position indicated by the comparison. (**B**) Arrangement of stimuli in the interval duration judgment experiment (Experiments 2–4). A clock with a blue hand and a fixation point at its center was presented. The hand showed 12 o’clock. Two horizontal green bars were flashed with a temporal separation of 100 ms (probe interval). A mask was presented at several times relative to the interval center. After a break the clock hand started turning for 48 ms to 144 ms (comparison interval). Subjects judged the durations of the probe and comparison interval and reported the shorter by pressing left or right arrow keys.

**Figure 2:**
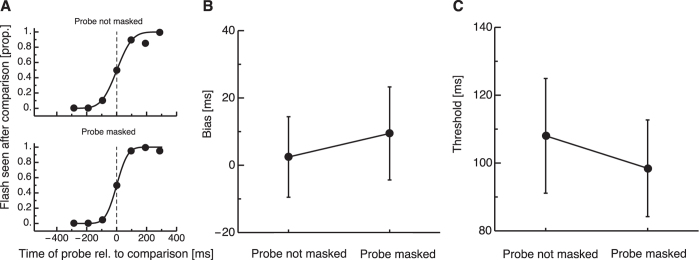
Results of the temporal order judgment experiment (Experiment 1). Error bars represent *SEM*. Data are shown for masked and unmasked probes. (**A)** Psychometric functions of a representative subject. The proportion of probes seen after the comparison is presented as a function of the time of the probe relative to the comparison. (**B**) Perceptual bias of masked and unmasked probe stimuli in milliseconds. (**C**) Average thresholds for the temporal registration of the probe in milliseconds.

We first asked whether the masking procedure - that marked the intervals in the main experiments - would change the temporal registration of the stimuli. In principle, a mask could change processing of the flashed stimuli such that they appeared to be earlier or later in time than stimuli that are not masked. To this end, we implemented a temporal order judgment task in which the time of flashed stimuli with and without mask had to be compared against the position of a clock-hand (see Fig. 1A). Please note, that in all experiments where stimuli were presented simultaneously with a mask, the stimuli were presented on top of the mask and were clearly visible. Flashed stimuli were presented at various times while a clock hand was moving. The clock hand started and stopped at its initial position after a full revolution (12 o’clock). Then, a comparison hand was shown and subjects had to judge whether the flashed stimulus was presented before or after the clock hand was in the position that the comparison hand is in. Thresholds were measured separately for stimuli presented together with a mask and stimuli without mask. Figure 2A shows psychometric functions from a representative subject. The upper panel shows data from trials in which the probe was not masked and the lower panel data from trials in which the probe was masked. For this subject there is virtually no bias in the temporal order judgements, neither when the probe is masked nor when it is not masked. Temporal order judgment bias averaged across all subjects is shown in Fig. 2B. Average bias for probes without mask was 2.47 ms (SEM 13.83 ms) and average bias for probes with mask 9.46 ms (SEM 11.98 ms). A paired t-test revealed no significant difference between the two conditions. We also wondered whether the mask could influence the precision for the temporal registration of the probe. Average thresholds are shown in Fig. 2C. Average thresholds for probes without mask were 108.02 (SEM 14.25 ms) and for probes with mask 98.45 ms (SEM 16.92 ms). A paired t-test revealed no significant difference between the two conditions. Temporal order judgments therefore did not provide any evidence that the mask significantly changed the temporal processing of the probe stimulus. To our own surprise, we observed no flash-lag effect, which clearly had to be expected in our temporal order judgment task^12^. We assumed that the reason for this might be the large width of the flashed bar we used in our task. We ran a control task with 4 subjects in which we shrunk the bar width to 5°. Under this condition we found a significant flash lag effect (22.08 ms (SEM 5.24 ms), p = 0.01). Since the flash-lag effect was not the focus of the present study, we continued our experiments with the original bar width of 17.5°.

We next asked how the mask would influence interval duration estimation when presented at different temporal positions in an interval. If changes in temporal interval judgements occur because the mask would bias the temporal registration of an interval marker, then different results would be expected if the mask is presented together with the first or the second interval marker. For instance, if the mask would delay probe registration then subjective interval duration should compress if the first interval marker is shown together with the mask. However, interval expansion should result if the mask is shown simultaneously with the second interval marker. In Experiment 2, two bars were flashed 100 ms after each other. The probe interval duration of 100 ms was constant in all trials.

**Figure 3:**
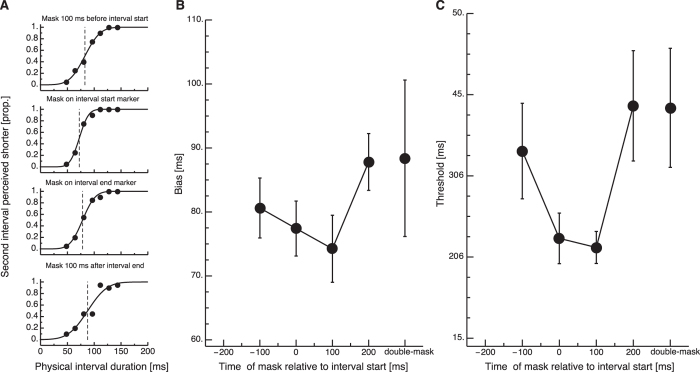
Results of the interval duration judgment experiment, passive (Experiment 2). (**A**) Psychometric functions from a representative subject. The proportion of the comparison interval perceived shorter than the probe is presented as a function of its physical interval duration in milliseconds. Data are shown for various positions of the mask relative to start marker of the probe interval (−100 ms, 0 ms, 100 ms, 200 ms). (**B**) Perceived duration of the first interval plotted for temporal position of the interval start marker relative to the mask. Error bars represent *SEM*.

Figure 3A shows psychometric functions from a representative subject. When the mask was presented 100 ms before onset of the interval start marker, the bias of this subject was close to the physical interval duration. However, when the mask was shown together with the first or the second marker the interval duration was underestimated. When the mask was shown after the interval end marker had been presented, duration estimation came closer to veridical again. Average interval duration judgments are shown in Fig. 3B. Intervals were judged to last 77.93 ms (SEM 5.37 ms) when the mask was shown 100 ms before the interval start marker. When the mask was shown simultaneously with the interval start or the interval end marker, interval duration was underestimated: When the interval start marker was shown simultaneously with the mask the interval appeared to last 74.89 ms (SEM 5.01 ms). When the interval end marker was shown simultaneously with the mask the interval appeared to last 68.78 ms (SEM 4.66 ms) ms. However, when the mask was shown after the probe interval, its duration was estimated to last 85.31 ms (SEM 5.08 ms). A oneway repeated measures ANOVA confirmed a significant main effect of interval duration estimation (F(3,27) = 6.22, p = 0.004). The mask thus produced temporal interval compression when either the interval start or end marker was presented together with a mask. However, there was also a general decrease in apparent interval duration. It is unlikely that the underestimation of the physical interval duration (100 ms) was produced by the mask which appeared 100 ms after the interval end marker. It might be related to time-order errors which have been observed in comparative judgments of duration^13^. Another factor is that the probe intervals were marked by flashed onsets whereas the comparison interval is marked by motion onsets. We also analyzed perceptual thresholds, which were given by the slope of the psychometric function. Average thresholds are shown in Fig. 3C. A time course very similar to the bias was observed for thresholds. Presenting the first or the second interval marker simultaneously not only induced compression, but also led to a reduction in thresholds. A oneway repeated measures ANOVA confirmed a significant main effect (F(3,27) = 3.859, p = 0.02). We also tested time estimations with two masks presented in each trial, one simultaneously with the first interval marker and the other simultaneously with the second interval marker. Average results from this condition are virtually identical to the condition in which the mask was shown 200 ms after the interval start marker. A paired t-test did not reveal a significant difference between these conditions.

**Figure 4:**
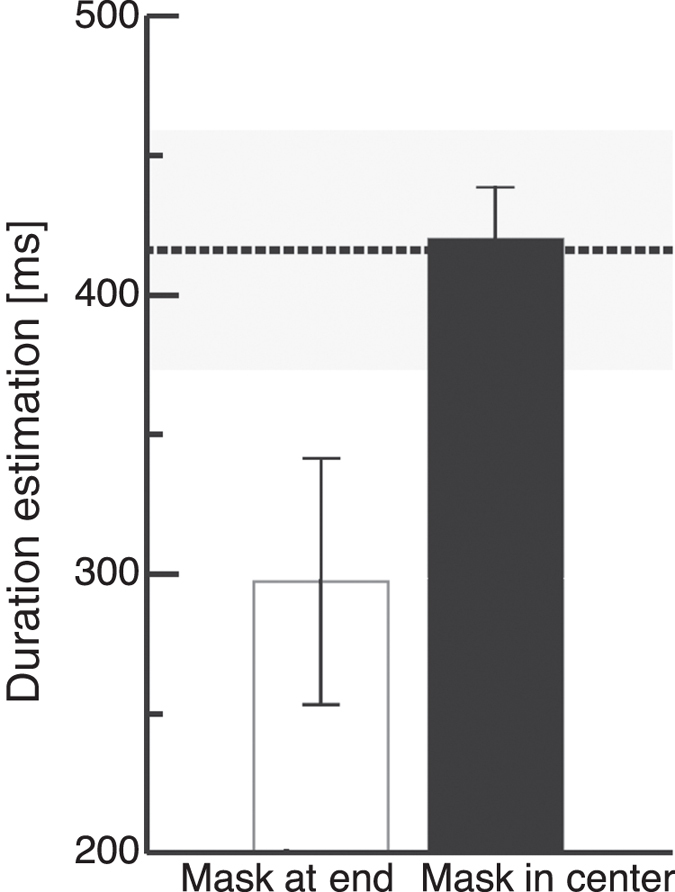
Results of the interval duration judgment experiment, with the mask in the temporal center (Experiment 4). Error bars represent *SEM*. The black dotted line marks averaged duration estimations for the baseline condition and the shaded area represents the standard error. A mask was presented simultaneously with the interval end marker or in the interval center.

If temporal binding is accomplished by the integration of interval markers with weak and strong onset signals, only masks presented in close temporal vicinity of the markers should lead to compression. To test this prediction we presented a mask in the interval center. In order to avoid that the mask was too close in time to marker presentation we chose an interval duration of 500 ms. A mask was presented either at the end of the interval, i.e. simultaneously with the interval end marker or in the temporal center of the interval. We presented trials with either no mask or with a mask in the temporal center of the interval between button press and appearance of the visual stimulus. Average interval duration estimations are shown in Fig. 4. The dashed line indicates the average baseline duration estimation and the shaded area its standard error. As in Experiment 1 underestimation of interval duration was observed in the baseline. When the mask was presented simultaneously with interval end marker, compression of interval duration occurred. However, as can be clearly seen, a mask shown in the interval center had no influence and average duration estimation was virtually identical to the baseline. A paired t-test confirmed a significant difference between temporal estimations with a mask at the interval end marking stimulus and a mask in the centre (t(6) = 2.59, p = 0.02). In a previous report^4^, we did find a compression effect with a mask in the interval center. Please note, that in this study the mask lasted 50 ms and was presented in a 100 ms interval. The mask therefore manipulated the onset strength of the second interval marker. In the present experiment the interval was 500 ms long and the mask was presented only for one frame (8 ms).

**Figure 5:**
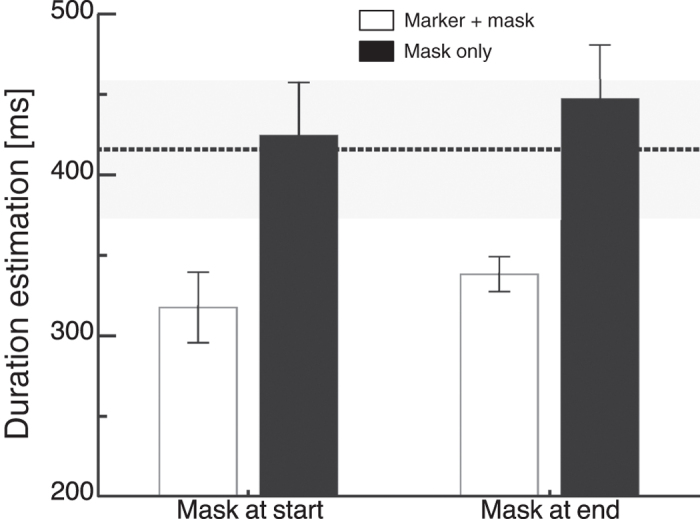
Results of the control condition. A mask is shown either at interval start or at interval end. In separate blocks the mask was shown either simultaneously with an interval marking stimulus or without any simultaneously presented stimulus. Depending on condition subjects either had to judge the interval duration between both interval markers or between mask and the remaining interval marker. Error bars represent *SEM*.

In all Experiments in which the mask is presented together with one of the interval markers, necessarily the mask is informative about interval start or end. Although explicitly instructed to judge the time passed between presentation of the interval markers, subjects could simply use the mask as a cue to report duration. In order to rule out this potential confound, we conducted a control Experiment. In this Experiment, subjects had to estimate the duration between either two interval markers or an interval marker and the mask itself. In both conditions, the mask was presented either at interval start or at interval end. If compression results from binding of corresponding interval makers, temporal estimations should remain veridical for intervals defined by a mask and a marker. Average result are shown in Fig. 5. The dashed line indicates again the average baseline duration estimation and the shaded area its standard error (same data as in Fig. 4). Bars shown in white represent trials in which intervals were defined by two marker stimuli. and a mask was presented additionally at interval start or end. Bars shown in black represent trials in which intervals were defined by a mask and a marker stimuli. We calculated a two-way repeated measures Anova with the factors “marker stimuli” (2 marker or 1 marker and the mask) and “mask position” (interval start/interval end). A significant main effect for the factor “marker stimuli” confirmed that temporal compression is significantly stronger when intervals were defined by two corresponding marker stimuli (F(1,5) = 9.985, p = 0.025).

**Figure 6:**
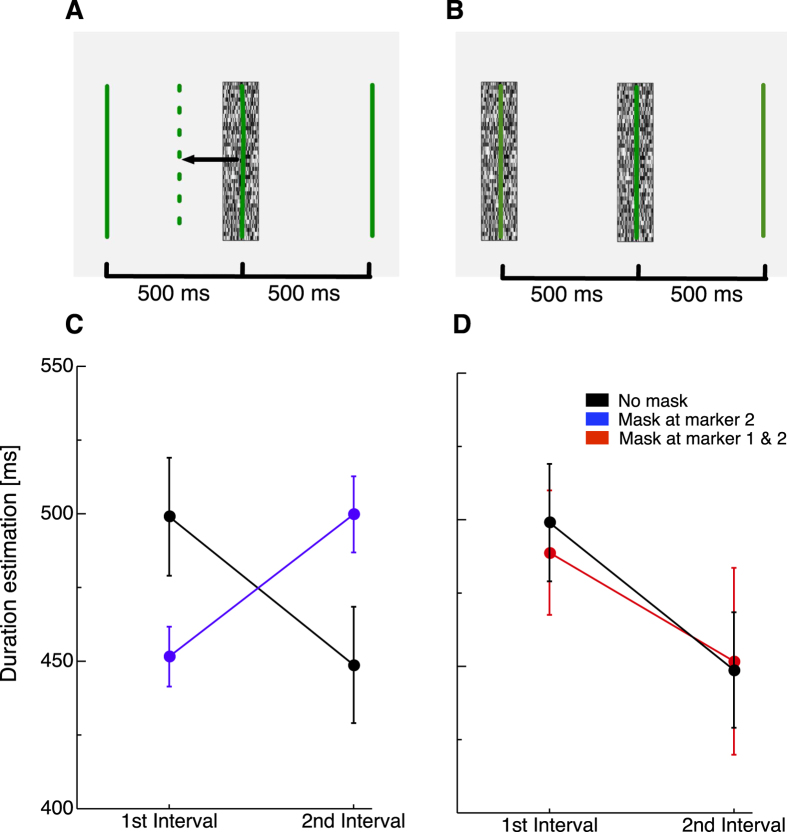
(**A**) Schematic illustration of the first experimental condition of Experiment 5. Three bars were presented sequentially, separated by two intervals, each lasting 500 ms. The second of the three bars was presented simultaneously with a whole-field mask as indicated by the mask symbol. As shift in time of the second bar towards the first was expected n this task, resulting in a compression of the first and an expansion of the second interval. (**B**) Schematic illustration of the second experimental condition of Experiment 5. Three bars were presented again sequentially, but the first and the second bar were presented together with a mask. According to the model view (see text) now compression was expected. (**C,D**) Results of the interval duration judgement experiment with two intervals (Experiment 5). Physical duration of both intervals was 500 ms. Error bars represent *SEM*. Black data points indicate average duration estimations for the baseline condition. Blue data points show average interval duration estimation when the second interval marker was presented simultaneously with a mask. Red data points show interval duration estimations when the first and the second interval marker shown simultaneously with a mask.

Finally, we aimed to test the idea of summing response distributions more explicitly. To this end, we presented three visual stimuli successively, marking two intervals, each with a physical duration of 500 ms. We first measured baseline duration estimations for both intervals without presenting any mask. Average baseline duration estimation is shown by the black data points in Fig. 6C,D. While the duration of the first interval was judged nearly veridically, the second interval duration was underestimated. We then presented the second stimulus in the sequence simultaneously with a mask (see Fig. 6A). Data from this condition are shown in blue. Masking the second stimulus led to a compression of the first and an expansion of the second interval. This result suggests a binding between first and second stimulus, which shrinks the first and dilates the second interval. In principle, binding could have occurred in both intervals, since Experiment 1 showed that both, masking interval start and end marker lead to compression. However, the mixed distribution representing the summation of first and second marker response distributions has its peak shifted in the direction of the peak representing the strong onset, i.e. the interval start marker, resulting in compression of the first interval.

If the second marker was bound to the first, necessarily then an expansion would be expected on the second interval. This is indeed what we found (see Fig. 6C). In a second step, we presented the three stimuli sequentially and now masked the first and the second marker (see Fig. 6B). The rationale behind this manipulation was that weakening the onset signal of the first stimulus should prevent a response distribution mixture of the first two markers and apparent duration should not be compressed. Data from this condition are shown in Fig. 6D in red color. As can be seen, duration estimations of both, the first and the second interval did hardly deviate from baseline judgments. The absence of compression in the first interval is consistent with the model view. A summation of distributions representing two weak onset signals should not shift the peak of the mixed distribution. The absence of expansion in the second interval seems to follow the absence of compression in the first. The data thus suggest that in a sequence of three stimuli the second stimulus is summed with the first and not to the third marker. A 3 × 2 repeated measures ANOVA with the factors “condition” (baseline/2nd stimulus masked/1st and 2nd stimulus masked) and intervals (1st/2nd) confirmed a significant interaction effect (F(2,10) = 6.48, p < 0.05).

## Discussion

**Figure 7:**
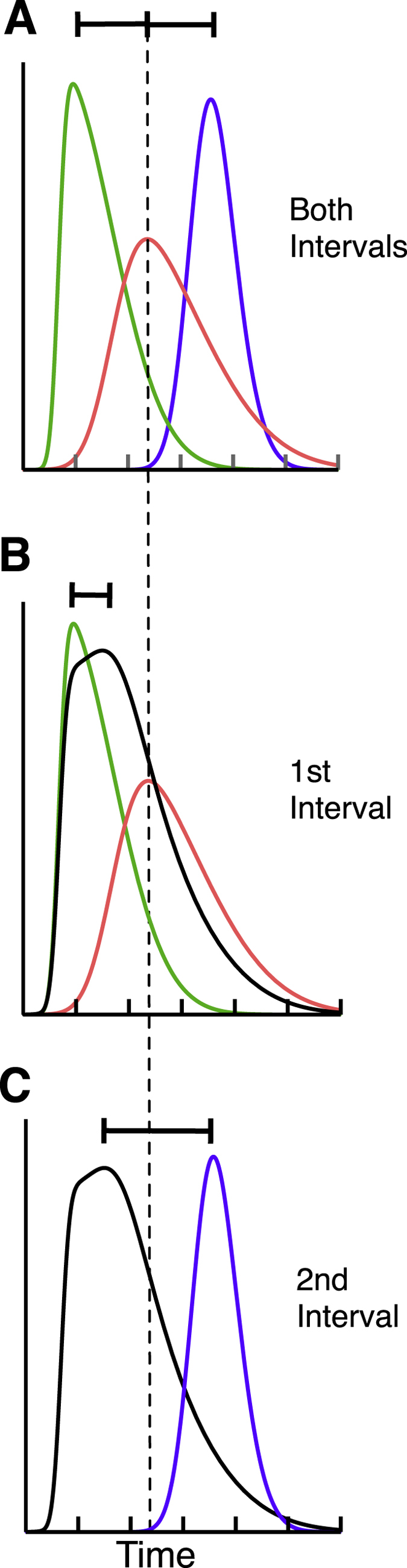
(**A**) Graphical sketch of the neural distributions responding to the first (shown in green), the second (shown in red) and the third (shown in blue) interval marker. The second interval marker is shown on top of a whole-field mask. Since the mask weakens the onset signal of the second interval marker, the corresponding neural response distribution is broader. (**B**) Summing the distributions corresponding to the first and the second interval marker results in a mixed distribution (shown in black) whose peak is shifted temporally in direction of the peak corresponding to the interval start marker. Reading off the interval duration from the peak of the first interval marker and the mixed distribution yields interval compression. (**C**) Since the mixed distribution is shifted into direction of the first interval marker, reading off the interval duration from the peak of the third interval marker and the mixed distribution yields interval expansion.

We have shown that interval duration estimations are biased by temporal binding between interval markers. Temporal binding occurred when the onset signal strength of one of the interval markers was artificially weakened by briefly flashing a whole-field mask. The mask had no influence when presented in the interval center but only when shown in close temporal vicinity of one of the markers (Experiment 4). Masking the interval start or the interval end marker both led to temporal binding, i.e. duration compression (Experiment 2). This rules out that changes in processing latencies for the masked marker are responsible for changes in apparent duration. Masking might change the apparent contrast^14^ or the amount of attention directed to the stimulus^15^. Both, contrast^16^,^17^,^18^ and attention^19^,^20^ are known to influence the temporal registration of stimuli. However in our temporal order judgement task we did not find evidence for a significantly different temporal registration of masked stimuli and stimuli without mask (Experiment 1). We explain temporal binding by a summation of the neural distributions responding to the marker with the weak and the marker with the strong onset signal. We assume that the peaks of these distributions represent the temporal occurrence of the marker stimulus (see Fig. 7). This information then is compared to a reference activity, either an explicit clock^7^ or implicit neural activation^8^, to produce an estimate of the time elapsed between the two markers. Strong onsets will induce distributions with a sharp peak and low variance (green line in Fig. 7A,B). Weak onsets however are reflected by broad distributions with a shallow peak. The peak of the mixed distribution (black line in Fig. 7B) would be shifted in the direction of the peak representing the strong onset, thus producing interval compression. Summing of the response distributions could be accomplished to minimize the variance of the final estimate. Indeed, we found that perceptual thresholds were significantly lower when intervals appeared compressed (Experiment 2). The mixed response distribution model predicts temporal compression only if one of the marker has a weak onset signal. If both marker have a weak onset signal, none of the peaks would shift. We have tested this prediction by presenting both of the marker simultaneously with a mask. The results confirmed the model prediction and no compression occurred (Experiment 2).

We explicitly tested this model by showing two intervals, marked by three visual stimuli presented sequentially. We then tested apparent duration of either the first or the second interval (Exp. 5). In the baseline condition we found that the first interval duration was estimated as longer as the second interval duration, as has been reported in a previous study^21^. A likely reason for this difference is that the first interval, unlike the second, appears with an abrupt onset^22^. Kanai *et al*.^22^ have shown that stimuli with an abrupt onset appear to last longer than stimuli with a motion onset. Since the interval markers appeared at different spatial positions, they generated the impression of apparent motion. Therefore the second interval, unlike the first, appeared with an apparent motion onset. Masking the second bar and thereby dampening its onset signal should lead to an underestimation of the first interval duration, as had been observed when only a single interval was presented (see Experiment 2). When the second interval marker was masked, the first interval appeared compressed and the second expanded. The expansion of the second interval duration is surprising, given that masking the first marker of an interval presented in isolation led to compression (see Experiment 2). We assume that subjects perceived the two intervals as a sequence and the expansion of the second interval followed consequently the compression of the first interval. In other words, if the second interval marker shifts in time towards the first, expansion of the second interval is the necessary implication. Thus, the direction of a temporal illusion - compression or expansion - also depends on the context in which intervals are presented. This result shows once more that temporal illusions follow functional principles to establish conference among stimuli, rather than being the result of variations in neural processing speed. We suggest that compression of the first and expansion of the second interval are the result of a response distribution summation of the first and second interval marker (see Fig. 7A,B). In this view, the second marker is shifted in time towards the first and away from the third (see Fig. 7B,C).

The expansion of the second interval is reminiscent of the “stopped clock illusion”^3^. In this illusion, a saccade performed to a clock induces an expansion of the interval immediately following the saccade. It has been demonstrated already that performing a saccade is only one condition amongst others to produce the effect^22^. As pointed out by Kanai *et al*.^22^ in all studies on this effect the first interval was always preceded by another stimulus^3^,^23^,^24^,^25^,^26^,^27^,^28^,^29^,^30^,^31^,^32^. Chronostasis thus might have the same origin as the expansion in our Experiment 5, that is a reduced onset signal of the second interval marker. Indeed, saccades decrease the apparent contrast of stimuli^33^ and they induce compression of time for intervals presented in isolation^1^. This interpretation implies the prediction that the first interval in chronostasis experiments - usually the interval between digit 0 and digit 1 -should appear compressed.

In conclusion, we suggest a principle of temporal binding in which interval markers with weak onset signals are attracted to interval markers with strong onset signals. If intervals are presented together with other intervals, temporal compression or expansion can occur, depending on the context. This mechanism minimizes uncertainty in the final estimate of interval duration, suggesting that temporal plasticity serves a functional role.

## Materials and Methods

### Participants

Ten subjects (5 female, 5 male, mean age 23 years) participated in Experiment 1 and 2. Seven different subjects (4 female, 3 male, mean age 24 years) participated in Experiment 3. Six different subjects (5 female, 1 male, mean age 30 years) participated in Experiment 4. Six different subjects (2 female, 4 male, mean age 26 years) participated in Experiment 5. All had normal or corrected to normal vision and were naive to the purpose of the experiment. Experiments were carried out in accordance with the Declaration of Helsinki. All experiments were approved by the local ethics committee of the psychological department of the Heinrich-Heine University Düsseldorf.

### Apparatus

Subjects were seated 57 cm from a Eizo FlexScan T57S. The visible screen diagonal was 20 inches, resulting in a visual field of 40 deg × 30 deg. Stimuli were presented on the monitor with a vertical frequency of 120 Hz on a homogeneously gray background.

### Experiment 1: Temporal Order Judgement

Subjects were required to keep fixation throughout the whole session at a fixation point (black, radius: 0.25°) which was presented in screen center. A circle (black, radius 5°) was presented in screen center throughout the whole session and served as the analogue of a clock without ticks. A blue bar (3.9° × 0.75°) mimicked the clock hand and was shown in the 12 ‘o clock position at trial start. After 1000 ms, the clock hand started turning clockwise for a full rotation that had a duration of 1136 ms and was effected in 71 equidistant steps of 16 ms. At variable times during the rotation a green horizontal probe bar (17.5° × 1.5°) was flashed 3.75° above the screen center for one frame. The bar could appear (at 16 equiprobable points in time) 320–800 ms after the hand started turning. After a full revolution of the clock hand was finished, the clock hand disappeared and 500 ms later, a red comparison hand appeared in the clock and remained visible until response. Subjects had to indicate the position where they saw the green clock hand at the time when the probe bar was flashed. They responded in a binary-forced-choice (2-AFC) task whether the green bar appeared to the left or to the right (in clockwise direction) of the comparison hand. They pressed the left or right arrow key of a normal computer keyboard. The comparison hand was presented in one of seven equiprobable spatial positions that represent a temporal range from 288 ms before the probe´s appearance to 288 ms thereafter. Two experimental sessions were conducted lasting 140 trials each. In one session the upper part of the screen was covered with a random-texture mask which was presented simultaneously with the probe bar. In the second session no mask was presented. The mask consisted of 40 × 30 rectangles (size: 1° × 1°) which each had a randomly assigned luminance on the gray scale level. In all Experiments the mask was presented for 1 frame (8 ms).

### Experiment 2: Interval duration estimation

A fixation point (black, radius: 0.25°) and a clock (black, radius 5°) were presented constantly throughout the whole session. After 1000 ms, a stimulus (green, 17.5° × 1.5°) was presented 5° above the screen center for one frame as the interval start marker. After a fixed interval of 100 ms (probe interval) the interval end marking stimulus (green, 17.5° × 1.5°) was presented 5° below the screen center. A whole screen random-texture mask was presented for one frame in each trial. The mask was presented in separate sessions either −100, 0, 100 or 200 ms relative to the presentation of the interval start marking stimulus. Thousand ms after interval end marking stimulus disappeared, the clock hand moved in clockwise direction for a duration chosen from 7 intervals (48 to 144 ms in seven equiprobable steps of 8 ms (comparison interval). After a full revolution, the clock hand remained in its current position until the next trial. In the next trial it started moving again where it had stopped in the last trial. Subjects indicated whether the probe or the comparison interval was shorter (2-AFC task) pressing the left or right arrow key of a normal computer keyboard with the index and middle finger of their right hand. There were 4 experimental sessions, each containing 140 trials. In separate sessions, we presented two masks per trial, one presented simultaneously with the first and one with the second interval marker.

### Experiment 3: Mask in interval center

Experiment 3 consisted of 3 conditions. The experimental details of these conditions were identical to those of Experiment 2, except that the interval duration was set to 500 ms and the temporal position of the mask differed. In condition 1, no mask appeared to measure the baseline performance, in condition 2, a mask was presented for one frame in the temporal center of the 500 ms interval and in condition 3 the mask was shown for one frame simultaneously with the interval end marking bar.

### Experiment 4: Control

Experiment 4 consisted of 2 × 2 conditions which were presented blockwise. A mask was presented either at interval start or end and the probe interval (500 ms duration) was defined either by two marker stimuli (identical characteristics as in Experiment 2) or by one marker stimulus and the mask itself. Depending on condition, subjects were instructed to compare the interval between the two marker stimuli or the interval between the mask and the remaining marker stimulus to the comparison interval. The comparison interval was implemented as in Experiment 2.

### Experiment 5: Interval duration estimation, two intervals

Subjects had to fixate on a black square (0.75° × 0.75°) which was visible throughout the trial. After a period of 500 ms, a horizontal green bar (17.5° × 1.5°) was presented 10° above screen center for one frame. After 500 ms a second horizontal green bar (17.5° × 1.5°) was presented at screen center for one frame. After additional 500 ms a third horizontal green bar (17.5° × 1.5°) was presented 10° below screen center for one frame. The second bar was presented on top of a whole-field random texture mask. After a period of 1000 ms, 2 bars were presented - each shown for one frame - separated by an interval with a duration that was varied across trials (comparison interval). Its duration ranged from 350 to 650 ms in steps of 50 ms. There were two experimental sessions, each consisting of 140 trials in which subjects were either instructed to estimate the first or the second interval duration and to compare it to the duration of the comparison interval. Stimulus positions in the comparison interval were identical to stimulus positions of the interval that had to be estimated. Thus, if subjects had to judge the first interval, markers of the comparison interval were presented 10° above the screen center and at the screen center. If the second interval had to be estimated, stimuli appeared at the screen center and 10° below the screen center.

## Additional Information

**How to cite this article**: Derichs, C. and Zimmermann, E. Temporal binding of interval markers. *Sci. Rep.* **6**, 38806; doi: 10.1038/srep38806 (2016).

**Publisher's note:** Springer Nature remains neutral with regard to jurisdictional claims in published maps and institutional affiliations.
